# Analysis of an ABE Scheme with Verifiable Outsourced Decryption

**DOI:** 10.3390/s18010176

**Published:** 2018-01-10

**Authors:** Yongjian Liao, Yichuan He, Fagen Li, Shaoquan Jiang, Shijie Zhou

**Affiliations:** 1 School of Information and Software Engineering, University of Electronic Science and Technology of China, Chengdu 610054, China; 201622060427@std.uestc.edu.cn (Y.H.); sjzhou@uestc.edu.cn (S.Z.); 2 School of Computer Science and Engineering, University of Electronic Science and Technology of China, Chengdu 611731, China; fagenli@uestc.edu.cn; 3 Institute of Information Security, Mianyang Normal University, Mianyang 621000, China; shaoquan.jiang@gmail.com

**Keywords:** attribute-based encryption, outsourced decryption, verifiable, cloud computing, authorized client, wireless sensor

## Abstract

Attribute-based encryption (ABE) is a popular cryptographic technology to protect the security of users’ data in cloud computing. In order to reduce its decryption cost, outsourcing the decryption of ciphertexts is an available method, which enables users to outsource a large number of decryption operations to the cloud service provider. To guarantee the correctness of transformed ciphertexts computed by the cloud server via the outsourced decryption, it is necessary to check the correctness of the outsourced decryption to ensure security for the data of users. Recently, Li et al. proposed a full verifiability of the outsourced decryption of ABE scheme (ABE-VOD) for the authorized users and unauthorized users, which can simultaneously check the correctness of the transformed ciphertext for both them. However, in this paper we show that their ABE-VOD scheme cannot obtain the results which they had shown, such as finding out all invalid ciphertexts, and checking the correctness of the transformed ciphertext for the authorized user via checking it for the unauthorized user. We first construct some invalid ciphertexts which can pass the validity checking in the decryption algorithm. That means their “verify-then-decrypt” skill is unavailable. Next, we show that the method to check the validity of the outsourced decryption for the authorized users via checking it for the unauthorized users is not always correct. That is to say, there exist some invalid ciphertexts which can pass the validity checking for the unauthorized user, but cannot pass the validity checking for the authorized user.

## 1. Introduction

Recently, cloud computing has become a very fascinating computing paradigm, in which storage and computation have moved away from terminal devices to the remote side. There are many novel applications in this area, such as outsourcing computation [1,2] and outsourcing verification [3]. This new and popular method brings important revolutions for the management, distribution and sharing data of enterprises and individuals, especially for some constrained devices, such as mobile phone, wireless sensors. Cloud clients (or sensors) are able to achieve significant cost savings by outsourcing their data storage and computation to some cloud service providers. Since the data of cloud clients (or sensors) are out of control by themselves, how to ensure the data security of cloud clients (sensors) is a significant problem in academia and industrial. Utilizing all kinds of cryptographic schemes is an essential method to achieve this goal. While attribute-based encryption (ABE) [4] is one of the most popular notions to study and utilize in cloud computing since it has the property of the flexible and fine-grained access control.

The notion of ABE was first introduced by Sahai and Waters [4]. There are two different types of ABE schemes according to the manner to deploy the access control policy, key-policy attribute-based encryption (KP-ABE) [5] and ciphertext-policy attribute-based encryption (CP-ABE) [6]. The ciphertexts are labeled with sets of attributes and access policies over these attributes are associated with clients’ private keys in the KP-ABE scheme. While every ciphertext is associated with an access policy, and every client’s private key is associated with a set of attributes in the CP-ABE scheme. However, decryption operations of most requirement that the set of attributes should satisfy the access policy in any ABE system and in most existing ABE schemes, one of the main drawbacks is that the length of the ciphertext and the decryption computational cost grow with the complexity of the access policy. This becomes critical obstacle in various applications, especially the applications on resource-limited devices.

In order to reduce the decryption time and the computation cost, Green et al. [7] proposed an ABE scheme with outsourced decryption (ABE-OD). In their scheme, an authorized client first delegated an untrusted cloud server to convert the original ciphertext into a transformed ciphertext with a transformation key, and then the client obtained the plaintext from the transformed ciphertext by spending a small overhead. The ABE-OD scheme would not leak any information about the encrypted data. However, the ABE-OD proposed by Green et al. cannot ensure the correctness of the transformed ciphertext since the cloud server is public and untrusted. The untrusted cloud server may send a wrong transformed ciphertext to the clients for saving computing cost or suffering from malicious attack which also causes to generate the incorrectly transformed ciphertext. In order to ensure the correctness of the ciphertext, Lai et al. [8] put forth an ABE-OD scheme that can check the correctness of the transformed ciphertext generated by the cloud server, which was called ABE with verifiable outsourced decryption (ABE-VOD). In their ABE-VOD scheme, the data owner encrypted a plaintext and a random message to the ciphertext respectively, and generated a commitment of an actual plaintext and the random message. And in the decryption algorithm of their ABE-VOD scheme, the client should compute the plaintext and the random message to use the commitment to verify whether the transformed ciphertext is generated correctly. A client was able to verify the correctness of the transformed ciphertext if and only if his/her attributes set satisfies the access structure associated with the ciphertext. Subsequently, several ABE-VOD schemes were proposed according to different methods and distinct scenarios in [9,10,11,12,13]. And Qiu et al. [14] used an ontology-based approach to achieve attribute-based access controls as well.

Recently, Li et al. [15] proposed a full verifiability for outsourced decryption in ABE, which could simultaneously check the correctness of transformed ciphertext for the authorized clients and unauthorized clients. In their scheme, a data owner constructed two access policies for the authorized clients and unauthorized clients, respectively. And then the data owner uses a short “signature” for each ciphertext to ensure that the client could verify the validity of the transformed ciphertext. In order to avoid first computing the plaintext and then verifying the validity of the ciphertext, Li et al. used “verify-then-decrypt” skill rather than “decrypt-then-verify” paradigm. That is to say, the client first verified the validity of the ciphertext or the transformed ciphertext, and then decrypted the ciphertext and obtains the corresponding plaintext or the random message if the ciphertext or the transformed ciphertext passed the verification of its validation.

### 1.1. Motivation and Contribution

In cloud computing, the ABE-OD scheme cannot ensure the correctness of the ciphertext or the transformed ciphertext for cloud server being untrusted. The untrusted server may send a wrong transformed ciphertext to the users for saving computing cost or it may have suffered from malicious attack which also produces the incorrect ciphertext or transformed ciphertext. In order to ensure the correctness of the ciphertext or the transformed ciphertext, the ABE-VOD schemes were proposed in [9,10,11,12,13,15].

However, we firstly show that the validity verification method in decryption algorithm of the ABE-VOD scheme put forth by Li et al. [15] cannot always check the validity of all ciphertexts in this paper. That is to say, there exist some invalid ciphertexts which can pass the validity checking and output the “corresponding” plaintexts. Furthermore, even if the untrusted server honestly performs the outsourced decryption for these invalid ciphertexts, the decryption algorithm cannot check them (the decryption algorithm cannot output ⊥). Thus, the “verify-then-decrypt” skill used in [15] is unavailable. Then, we show that the method to check the validity of the outsourced decryption for the authorized user via checking it for the unauthorized user is not always correct. That is to say, there exist some invalid ciphertexts which can pass the validity checking for the unauthorized user, but cannot pass the correctness of the ciphertexts checking for the authorized user.

### 1.2. Organization of the Paper

The rest of this paper is organized as follows. The system model of the ABE-VOD and some basic mathematic knowledge are introduced in Section 2. In Section 3, we review the ABE-VOD scheme proposed by Li et al., and analyze their scheme. Finally, the conclusions are given in Section 4.

## 2. Premilinary

In the section, we will recall the definition of ABE-VOD and some basic mathematic knowledge in [15].

### 2.1. System Model

The ABE-VOD Scheme consists of seven algorithms: Setup, KeyGen, Encrypt, Decrypt, GenTK${}_{out},$ Transform${}_{out}$ and Decrypt${}_{out}.$ The detailed is described as follows.

- Setup$(1^{\lambda },U).$ Take as input a security parameter $1^{\lambda }$ and attribute universe description U, generate a master secret key msk and public parameters PK.
- KeyGen(msk,PK,S). Take as input the master secret key msk, the public parameters PK and an attribute set S, generate the client’s private key SK. If a client is an authorized one, use $SK_{DS}$ to represent the private key of the authorized client, where DS represents an attribute set of the authorized client. If a client is an unauthorized one, the client uses $SK_{VS}$ to represent the private key of the unauthorized client, where VS represents an attribute set of the unauthorized client.
- Encrypt$(PK,M,A,\bar{A}).$ Take as input the public parameters PK, the plaintext *M* and two access structures $A,\bar{A},$ and output a ciphertext CT.
- Decrypt(SK,CT). Take as input a private key SK and a ciphertext CT. If the client’s attribute set *S* satisfies the access policy A, then the client utilizes the private key $SK_{DS}$ to decrypt the ciphertext; otherwise, the client utilizes the private key $SK_{VS}$ to decrypt the ciphertext. After the client checks the correctness of the ciphertext, he/she outputs the plaintext *M* if the ciphertext is valid; otherwise, the client outputs ⊥.
- GenTK${}_{out}\left(PK,SK\right).$ Take as input the public parameters PK and the private key SK, genetate a transformation key TK and a retrieving key RK. If a client is an authorized one, let $SK=SK_{DS}$ and set $TK=TK_{DS},$ $RK=RK_{DS};$ otherwise, let $SK=SK_{VS}$ and set $TK=TK_{VS},$ $RK=RK_{VS}.$
- Transform${}_{out}\left(TK,CT\right).$ Take as input the transformation key TK and the ciphertext CT, generate the transformed ciphertext TCT.
- Decrypt${}_{out}\left(CT,TCT,RK\right).$ Take as input a ciphertext CT, a transformed ciphertext TCT and a retrieving key RK. If the client’s attribute set *S* satisfies the access policy A, the client is an authorized one and then he/she utilizes CT, TCT and $RK_{DS}$ to decrypt the ciphertext; otherwise, the client utilizes the private key CT, TCT and $RK_{VS}$ to decrypt the ciphertext. After the client checks the correctness of the ciphertext, outputs the plaintext *M* if the ciphertext is valid; otherwise, outputs ⊥.

### 2.2. Bilinear Pairing

Let $G_{1}$ and $G_{2}$ be two multiplicative groups which have the same prime order q, $Z_{q}^{*}$ be the multiplicative group of the finite field $F_{q}.$ A bilinear map $e:G_{1}\times G_{1}\to G_{2}$ [16], which satisfies the followings three properties:

- Bilinearity: For any $\alpha ,\beta ,\gamma \in G_{1},$
  e(α,βγ)=e(α,β)e(α,γ),and
  e(αβ,γ)=e(α,γ)e(β,γ).
- Non-degeneracy: There are elements $\alpha ,\beta \in G_{1},$ such that e(α,β)≠1, where 1 is the identity element of $G_{2}$.
- Computability: For any elements $\alpha ,\beta \in G_{1},$ there is an efficient algorithm to compute e(α,γ).

The concrete bilinear pairings *e* will be using the modified Weil [17] or Tate pairings [18] on some elliptic curves. We will define two hard problems used in our paper below: Decisional Diffie-Hellman (DDH) problem and Computational Diffie-Hellman (CDH) problem. Let α be a generator of the group $G_{1}.$

**Definition** **1.** (CDH problem in $G_{1}).$ Given $\alpha ,\;\alpha^{x},\;\alpha^{y}\in G_{1}$, to compute $\alpha^{xy}.$

**Definition** **2.** (DDH problem in $G_{1}).$ Given α,$\alpha^{x},$$\alpha^{y},$$\alpha^{z}$$\in G_{1}$, to decide whether xy≡zmodq holds or not.

It is obvious that the DDH problem in $G_{1}$ is easy since it can verify above congruence by using the bilinear pairing *e*. However, as far there is no polynomial-time algorithm to solve CDH problem in $G_{1}$, we assume that CDH problem in $G_{1}$ is hard.

### 2.3. Linear Secret Sharing Schemes

We recall a description for LSSS in [19]. Let P be a set of parties. A secret sharing scheme Π is called linear (over $Z_{p}$) if it satifies the following conditions.

- The secret shares of each party form a vector in $Z_{p}.$
- Let *A* is a matrix with *l* rows and *n* columns. Let the function ρ represent the party labeling row *i* as ρ(i), where is the *i*th row of A. Suppose a vector ${\vec{v}}_{i}={\left(s,r_{2},\ldots ,r_{n}\right)}^{T}$ is the column vector and $r_{2},\ldots ,r_{n}$ are random value in $Z_{p},$ where $s\in Z_{p}$ is the secret to be shared. $A\vec{v}$ is the vectors of *l* shares for the the secret *s* with respect to Π. The share ${\left(A\vec{v}\right)}_{i}$ belongs to party ρ(i). Suppose that Π is an LSSS of the access policy A and S∈A is any authorized set. Let I={i:ρ(i)∈S}⊂[l]={1,2,…,l}. If $\left\{\lambda_{i}\right\}$ are valid shares for any secret *s* with respect to Π, then we can compute constants ${\left\{\omega_{i}\in Z_{p}\right\}}_{i\in I}$ such that $\sum_{i\in I}\omega_{i}\lambda_{i}=s,$ where $\lambda_{i}={\left(A\vec{v}\right)}_{i}.$

Notations. The vector (1,0,…,0) is the “target” vector of any LSSS. For any unauthorized set of rows *I* in A, the target vector is not in the span of the rows of set I. For any authorized set of rows *I* in A, the target vector is in the span of I.

## 3. Analysis of Li et al.’s Abe-Vod Scheme

Since ABE-VOD scheme proposed by Li et al. is much complex, we recall it in Appendix B and the security model in Appendix A.

### 3.1. The Excepted Functionalities of the ABE-VOD Scheme

In the subsection, we analyze the construction of the ABE-VOD scheme proposed by Li et al. The scheme wanted to get the following results at least.

- First, any ABE-VOD should have the decryption functionality. The decryption algorithm of the ABE-VOD can correctly check the valid ciphertext and invalid ciphertext (any encryption scheme must satisfy this condition). That is to say, the Decrypt algorithm outputs a corresponding plaintext of some ciphertext if and only if the ciphertext is valid, or the Decrypt${}_{out}$ algorithm outputs the corresponding plaintext of a transformed ciphertext if and only if the transformed ciphertext is correct.
- Then, the ABE-VOD scheme can simultaneously check the correctness of the transformed ciphertext for the authorized users and unauthorized users by using “verifying-then-decrypt” method to guarantee the correctness of the transformed ciphertext.

### 3.2. The ABE-VOD Scheme Cannot Verify the Validity of All Ciphertexts

In general, the goal of the verification formulas of the decryption algorithm are to check the correctness of ciphertext. However, the decryption algorithm of ABE-VOD scheme proposed by Li et al. only checks validity of a part of ciphertext, but not checks whether the output of the decryption algorithm for some ciphertext is the original plaintext . In the subsection, we show that there exist some ciphertexts which are verified by the decryption algorithm, but its output isn’t the original plaintext.

As analysis in [15], the ciphertext stored in cloud server maybe be tampered by some malicious attackers or the transformed ciphertext could be generated via using incorrect one by the untrusted cloud server. We will view these activities as attacks of an adversary and describe how an adversary constructs an invalid ciphertext below, which the decryption algorithm will view as a valid ciphertext and output the “corresponding” plaintext.

The adversary takes as input a random message $M\in {\left\{0,1\right\}}^{m}$ and the two LSSS access structures A = (A,ρ), $\bar{A}$ = $(\bar{A},\bar{\rho }).$

The adversary first picks up a random string $R\in {\left\{0,1\right\}}^{m},$ two random vectors

$$\vec{v}=\left(s_{1},v_{12},\cdots ,v_{1n}\right)\in {\left(Z_{p}^{*}\right)}^{n}$$

and

$${\vec{v}}^{\prime }=\left(s_{2},v_{22},\cdots ,v_{2n}\right)\in {\left(Z_{p}^{*}\right)}^{n}$$

and two random elements $s_{1}^{\prime },s_{2}^{\prime }\in Z_{p}^{*}$ such that $s_{1}\neq s_{1}^{\prime }$ and $s_{2}\neq s_{2}^{\prime }.$ For each row $A_{i}$ of *A*, ${\bar{A}}_{i}$ of $\bar{A}$, it picks $r_{1,i},r_{2,i}\in Z_{p}^{*}$ uniformly at random. Then, it calculates:

$$C_{M}=M⊕H_{3}\left(e{\left(g,g\right)}^{\alpha s_{1}^{\prime }}\right),$$

$$\eta_{1}=H_{1}\left(e{\left(g,g\right)}^{\alpha s_{1}}\right),$$

$$C_{1}=g^{s_{1}},$$

$$C_{1,i}=g^{aA_{i}\cdot \vec{v}}T_{\rho (i)}^{-r_{1,i}},D_{1,i}=g^{r_{1,i}}\forall i\in \left\{1,2,\cdots ,l\right\}.$$

Set $CT_{M}=\left(C_{M},C_{1},{\left\{C_{1,i}\right\}}_{i\in [l]},{\left\{D_{1,i}\right\}}_{i\in [l]}\right),$ and compute:

$$C_{R}=R⊕H_{3}\left(e{\left(g,g\right)}^{\alpha s_{2}^{\prime }}\right),$$

$$\eta_{2}=H_{1}\left(e{\left(g,g\right)}^{\alpha s_{2}}\right),$$

$$C_{2}=g^{s_{2}},$$

$$C_{2,i}=g^{a{\bar{A}}_{i}\cdot {\vec{v}}^{\prime }}T_{\bar{\rho }\left(i\right)}^{-r_{2,i}},D_{2,i}=g^{r_{2,i}},\;\forall i\in \left[l\right].$$

Set $CT_{R}=\left(C_{R},C_{2},{\left\{C_{2,i}\right\}}_{i\in [l]},{\left\{D_{2,i}\right\}}_{i\in [l]}\right).$

$$\sigma_{1}=H_{2}{\left(C_{M},C_{R}\right)}^{\eta_{1}},\sigma_{M}=\left\{\sigma_{1},H_{2}\left(C_{M},C_{R}\right)\right\},$$

$$\sigma_{2}=H_{2}{\left(C_{M},C_{R}\right)}^{\eta_{2}},\sigma_{R}=\left\{\sigma_{2},H_{2}\left(C_{M},C_{R}\right)\right\}.$$

The ciphertext $CT=(A,\bar{A},CT_{M},\sigma_{M},CT_{R},\sigma_{R}).$

Obviously, the ciphertext CT is not a valid ciphertext of the message *M* since the adversary picks two distinct random numbers $s_{1}$ and $s_{1}^{\prime }$ to produce the ciphertext $CT_{M}$, and picks two distinct random numbers $s_{2}$ and $s_{2}^{\prime }$ to produce the ciphertext $CT_{R}$. However, the decryption algorithm will view it as a valid ciphertext and output the “corresponding” plaintext. When the decryption algorithm takes as input CT and SK, it runs as follows.

- If *S* satisfies the access policy A, the private key SK of an authorized client is
  $$(DS,K=g^{\alpha }y^{t_{1}},K_{0}=g^{t_{1}},{\left\{K_{i}=T_{i}^{t_{1}}\right\}}_{att_{i}\in DS}).$$
  Let I={i:ρ(i)∈S}⊂[l]={1,2,⋯,l}. Then it calculates $\omega_{i}\in Z_{p}^{*}$ for i∈I such that $\Sigma_{i\in I}\omega_{i}A_{i}$ = (1,0,⋯,0), and computes:
  $$X_{M}=\frac{e(C_{1},K)}{\prod_{i\in I}{\left(e\left(C_{1,i},K_{0}\right)e\left(D_{1,i},K_{\rho (i)}\right)\right)}^{\omega_{i}}},$$
  which equals $e{\left(g,g\right)}^{\alpha s_{1}}.$
  It is clear that the equality
  $$e\left(\sigma_{1},g\right)=e(H_{2}(C_{M}\left|\right|C_{R}),g^{\eta_{1}})$$
  holds, where $\eta_{1}=H_{1}\left(X_{M}\right).$ Then it computes
  $$M^{\prime }=C_{M}⊕H_{3}\left(X_{M}\right)=M⊕e{\left(g,g\right)}^{\alpha s_{1}^{\prime }}⊕e{\left(g,g\right)}^{\alpha s_{1}}.$$
  However, $M^{\prime }$ does not equal *M* since $s_{1}^{\prime }\neq s_{1}.$ That is to say, the decryption algorithm cannot refuse the plaintext of the ciphertext which is produced by other “encryption” algorithm.
- If *S* satisfies the access policy $\bar{A}$, the private key SK of an unauthorized client is
  $$(VS,K=g^{\alpha }y^{t_{2}},K_{0}=g^{t_{2}},{\left\{K_{i}=T_{i}^{t_{2}}\right\}}_{att_{i}\in VS}).$$
  Let $I=\{i:\bar{\rho }\left(i\right)\in S\}\subset \left\{1,2,\cdots ,l\right\}.$ Then it calculates $\omega_{i}\in Z_{p}^{*}$ for i∈I such that $\Sigma_{i\in I}\omega_{i}\bar{A_{i}}$ = (1,0,⋯,0), and computes:
  $$X_{R}=\frac{e(C_{2},KP)}{\prod_{i\in I}{\left(e\left(C_{2,i},KP_{0}\right)e\left(D_{2,i},KP_{\bar{\rho }\left(i\right)}\right)\right)}^{\omega_{i}}},$$
  which equals $e{\left(g,g\right)}^{\alpha s_{2}}.$
  For the same reason above, the equality
  $$e\left(\sigma_{2},g\right)=e(H_{2}(C_{M}\left|\right|C_{R}),g^{\eta_{2}})$$
  holds, where $\eta_{2}=H_{1}\left(X_{R}\right).$ Then it computes
  $$R^{\prime }=C_{R}⊕H_{3}\left(X_{R}\right)=R⊕e{\left(g,g\right)}^{\alpha s_{2}^{\prime }}⊕e{\left(g,g\right)}^{\alpha s_{2}}.$$
  However, $R^{\prime }$ does not equal *R* since $s_{2}^{\prime }\neq s_{2}.$

Thus, the decryption algorithm of the ABE-VOD scheme proposed by Li et al. for both the authorized client and the unauthorized client cannot check the validity of all ciphertexts. I.e., there exist some invalid ciphertexts which can pass the validity checking. Furthermore, their ABE-VOD scheme cannot check the validity of the outsourcing computation by checking the correctness of the corresponding ciphertext since the output of both the Decrypt algorithm and Decrypt${}_{out}$ algorithm is not always correct.

### 3.3. The ABE-VOD Scheme Is Not Full Verifiable

Since verifying the correctness of the outsourced decryption for unauthorized clients is very important, Li et al. considered the following scenario. The authorized user wants to, but is not able to, process some pending businesses when the time or position of the authorized client is limited. He/she needs someone to help him/her to verify whether a pending business is correctly processed and does not want the latter to know anything about the content of the business. Thus Li et al. proposed the ABE-VOD scheme which could utilize an unauthorized client to help him/her to verify the correctness of the transformed ciphertext. We construct the following ciphertext which can pass the correctness checking for an unauthorized client but it is not a valid ciphertext for the authorized client.

The adversary takes as input a random message $M\in {\left\{0,1\right\}}^{m}$ and the two LSSS access structures A = (A,ρ), $\bar{A}$ = $(\bar{A},\bar{\rho }).$

The adversary first picks a random string $R\in {\left\{0,1\right\}}^{m},$ two random vectors

$$\vec{v}=\left(s_{1},v_{12},\cdots ,v_{1n}\right)\in {\left(Z_{p}^{*}\right)}^{n}$$

and

$${\vec{v}}^{\prime }=\left(s_{2},v_{22},\cdots ,v_{2n}\right)\in {\left(Z_{p}^{*}\right)}^{n}.$$

For each row ${\bar{A}}_{i}$ of $\bar{A}$, it picks $r_{2,i}\in Z_{p}^{*}$ uniformly at random. And it uniformly picks

$$C_{M}\in {\left\{0,1\right\}}^{m},\;\eta_{1}\in Z_{p}^{*},\;C_{1},\;{\left\{C_{1,i},\;D_{1,i}\right\}}_{\left[l\right]}\in G_{1}$$

at random.

Set $CT_{M}=\left(C_{M},C_{1},{\left\{C_{1,i}\right\}}_{i\in [l]},{\left\{D_{1,i}\right\}}_{i\in [l]}\right)$, then it calculates:

$$C_{R}=R⊕H_{3}\left(e{\left(g,g\right)}^{\alpha s_{2}}\right),$$

$$\eta_{2}=H_{1}\left(e{\left(g,g\right)}^{\alpha s_{2}}\right),$$

$$C_{2}=g^{s_{2}},$$

$$C_{2,i}=g^{a{\bar{A}}_{i}\cdot {\vec{v}}^{\prime }}T_{\bar{\rho }\left(i\right)}^{-r_{2,i}},D_{2,i}=g^{r_{2,i}},\;\forall \;i\in \left[l\right].$$

Set $CT_{R}=\left(C_{R},C_{2},{\left\{C_{2,i}\right\}}_{i\in [l]},{\left\{D_{2,i}\right\}}_{i\in [l]}\right).$

$$\sigma_{1}=H_{2}{\left(C_{M},C_{R}\right)}^{\eta_{1}},\sigma_{M}=\left\{\sigma_{1},H_{2}\left(C_{M},C_{R}\right)\right\},$$

$$\sigma_{2}=H_{2}{\left(C_{M},C_{R}\right)}^{\eta_{2}},\sigma_{R}=\left\{\sigma_{2},H_{2}\left(C_{M},C_{R}\right)\right\}.$$

The ciphertext $CT=(A,\bar{A},CT_{M},\sigma_{M},CT_{R},\sigma_{R}).$

It is clear that if *S* satisfies the access policy A, the authorized client cannot pass the checking of the correctness of the ciphertext. Because the elements $C_{M},C_{1},{\left\{C_{1,i}\right\}}_{i\in [l]},{\left\{D_{1,i}\right\}}_{i\in [l]}$ are random elements, which is a valid ciphertext with a negligible probability. That is to say, since the equation $\eta_{1}=H_{1}\left(\frac{e(C_{1},K)}{\prod_{i\in I}{\left(e\left(C_{1,i},K_{0}\right)e\left(D_{1,i},K_{\rho (i)}\right)\right)}^{\omega_{i}}}\right)$ with negligible probability for random elements $C_{M},$ $C_{1},$ $\eta_{1},$ ${\left\{C_{1,i}\right\}}_{i\in [l]},$ ${\left\{D_{1,i}\right\}}_{i\in [l]},$ $\sigma_{1}$ is a valid signature of $H_{2}\left(C_{M},C_{R}\right)$ with negligible probability. We use the decryption algorithm to check the equality

$$e\left(\sigma_{1},g\right)=e(H_{2}(C_{M}\left|\right|C_{R}),g^{\eta_{1}}),$$

which holds with negligible probability for random elements $C_{M},C_{1},$ $\eta_{1},$ ${\left\{C_{1,i}\right\}}_{i\in [l]},{\left\{D_{1,i}\right\}}_{i\in [l]}.$

However, if *S* satisfies the access policy $\bar{A},$ the unauthorized client can pass the correctness checking of the ciphertext. Because the adversary uses the Encrypt algorithm to encrypt the message *R* for the unauthorized client. The equations

$$\eta_{2}=H_{1}\left(e{\left(g,g\right)}^{\alpha s_{2}}\right),$$

$$\mathrm{and}\;\sigma_{2}=H_{2}{\left(C_{M},C_{R}\right)}^{\eta_{2}}$$

hold. That means

$$e\left(\sigma_{2},g\right)=e(H_{2}(C_{M}\left|\right|C_{R}),g^{\eta_{2}})$$

always holds. Thus, the decryption algorithm can output plaintext *R* correctly. Especially, when the untrusted server honestly runs the Transform${}_{out}$ algorithm, the unauthorized client can always pass the correctness checking of the transformed ciphertext.

Thus, the ABE-VOD scheme cannot verify the correctness of the ciphertext or the transformed ciphertext for the authorized user via verifying it for the unauthorized user.

### 3.4. Furthermore Analysis

We have showed that the decryption algorithm cannot satisfy two functionalities, checking the correctness of all ciphertexts and “full verifiable” above. Next, we will explain the reason and possibly reasonable method.

On one hand, the construction of the above ABE-VOD scheme utilized ABE-OD scheme proposed by Green et al. [7] and short signature scheme proposed by Boneh et al. [16]. The one-time signature $\sigma_{1}$ of a “message” $H_{2}\left(C_{M},C_{R}\right)$ (or $\sigma_{2}$ of a “message” $H_{2}\left(C_{M},C_{R}\right)$) is unforgeable and it also ensures that

$$e\left(\sigma_{1},g\right)=e(H_{2}(C_{M}\left|\right|C_{R}),g^{\eta_{1}})$$

or

$$e\left(\sigma_{2},g\right)=e(H_{2}(C_{M}\left|\right|C_{R}),g^{\eta_{2}})$$

holds if and only if $\sigma_{1}$ and $\sigma_{2}$ are valid signatures of $H_{2}(C_{M}\left|\right|C_{R})$ (or $C_{M}$ and $C_{R}$) under public key $g^{\eta_{1}}$ and $g^{\eta_{2}},$ respectively. However, there is no condition that guarantees the validity of $C_{M}$ and $C_{R}.$ That is to say, we can choose any random element as $C_{M}$ (or $C_{R}$). Thus, the above adversary can construct an invalid $C_{M}$ or $C_{R}$ but the ciphertext CT can be verified as a valid ciphertext. It seems that the method to sign a part of the ciphertext cannot guarantee all invalid ciphertexts to be refused. It needs another secure mechanism to guarantee the part of the ciphertext is valid.

On the other hand, from the unauthorized client’s view, $C_{M}$ is a random element in ${\left\{0,1\right\}}^{m},$ which is independent of $C_{R},\bar{A}$ and $\sigma_{R}.$ Thus, the unauthoized client has no capability to verify the validity of $C_{M},$ and the construction in [15] cannot check the correctness of the ciphertext and the transformed ciphertext for the authorized users by checking the validity of the ciphertext and the correctness of the transformed ciphertext for the unauthorized clients.

## 4. Conclusions

In this paper, we showed that the validity verification method in decryption algorithm of the ABE-VOD scheme put forth by Li et al. cannot always check the validity of all ciphertexts. There exist some invalid ciphertexts which can pass the validity checking and the “verify-then-decrypt” skill used in [15] is unavailable. Then, we showed that the method to check the validity of the outsourced decryption for the authorized client via checking it for the unauthorized client was not always correct. There exist some invalid ciphertexts which can pass the validity checking for the unauthorized client but cannot pass the validity checking for the authorized client. Finally, we pointed out that although the scheme used signature skill to guarantee the ciphertext cannot be tampered, the signing key of the “signature scheme” used in the encryption scheme was not fixed and anyone can generated it. That caused our constructions.

## Appendix A. Security Model

We recall the security model in [15]. We first consider the selective chosen plaintext attack (CPA) security model for ABE with fully verifiable outsourcing decryption is described by the following game between an adversary A and a challenger C.

- Init. The adversary A sets a challenge access policy $A^{*}$ that it wishes to challenge.
- Setup. The challenger C executes the algorithm Setup to generate the public parameters PK and the master secret key msk. C sends PK to A, and keeps msk secret.
- Phase1. The challenger C sets a set *D* and a table *T* initially empty. The adversary A makes the following queries:
  - (1) Privatekey query. The adversary A makes private key queries on an attribute set S, the challenger C runs KeyGen algorithm to generate a private key $SK_{S},$ and sets D=D∪S. Then it returns the private key to the adversary A. The only restriction is that the attribute set cannot satisfy the access policy $A^{*}.$
  - (2) Transformationkey query. A makes transformation key queries on an attribute set S, and C searches the tuple $\left(S,SK_{S},TK_{S},RK_{S}\right)$ in the table T. If such tuple exists, it returns $TK_{S}$ as response. Otherwise, it executes KeyGen(PK,msk,S) to generate $SK_{S}$ and GenTK${}_{out}\left(PK,SK_{S}\right)$ to generate $(TK_{S},RK_{S}).$ Then the adversary A stores the tuple $\left(S,SK_{S},TK_{S},RK_{S}\right)$ in table T. It returns the transformation key $TK_{S}$ to A.
- Challenge. The adversary A submits two messages $M_{0}$ and $M_{1}$ with the same size. Then C randomly picks a bit b∈{0,1} and *R* with the same length as $M_{0}$ and $M_{1},$ and computes $CT^{*}=$ Encrypt$\left(PK,M_{b},A^{*}\right)$. Finally, the challenger C sends to $CT^{*}$ to A as a challenge ciphertext.
- Phase2. A proceeds to make Privatekey queries and Transformationkey queries as Phase 1, however the only restriction is that the attribute set does not satisfy the access policy $A^{*}.$
- Guess. A outputs its guess $b^{\prime }\in \left\{0,1\right\}$ with respect to *b* and wins the game if $b^{\prime }=b.$

The advantage of the adversary A in the above game is

$$|\mathrm{Pr}\left[b^{\prime }=b\right]-\frac{1}{2}|,$$

where the probability is taken over the random bits by the adversary A and the challenger C.

**Definition** **A1.** An ABE-VOD scheme is selective CPA-secure if every polynomial time adversary A has at most a negligible advantage in the above game.

Next, we review the formal definition of *verifiability* for an ABE-VOD scheme through a game between an adversary A and a challenger C [15]. The definition is just considered the part of the authorized user here, which is the same as the definition of verifiability for the unauthorized user. The game is described as follows:

- Init. The adversary A sets an access policy $A^{*}$ that it wishes to challenge.
- Setup. The challenger runs the Setup$(1^{\lambda },U$) to generate the public parameters PK and the master key msk, then keeps msk secret and sends PK to the adversary.
- Phase1. The adversary A can execute the privatekey query and the transformationkey query as in Phase 1 in the above security game.
  - (1) Privatekey query. The adversary A makes private key queries on an attribute set S, the challenger runs KeyGen(msk,PK,S) to generate SK and sets D=D⋃{S} which is initially empty. It then returns the private key $SK_{S}$ to the adversary. The only restriction is that the attribute set *S* cannot satisfy the access policy $A^{*}.$
  - (2) Transformationkey query. A makes transformation key queries on the attribute set S; C searches the tuple (S, $SK_{S},$ $TK_{S},$ $RK_{S})$ in the table T. If the tuple exists, C returns $TK_{S}$ as a response. Otherwise, it executes KeyGen(msk, PK, S) to generate $SK_{S}$ and GenTK${}_{out}(PK,$ $SK_{S})$ to generate $(TK_{S},$ $RK_{S}).$ Then C stores the tuple (S, $SK_{S},$ $TK_{S},$ $RK_{S})$ in table *T* and returns the transformation key $TK_{S}$ to A.
- Challenge. The adversary submits a message $M^{*}.$ The challenger computes a challenge ciphertext $CT^{*}$ = Encrypt$\left(PK,M^{*},A^{*}\right)$ and sends it to A.
- Phase2. The same as Phase 1.
- Output. The adversary outputs an attributes set $S^{*}$ and a transformed ciphertext $TCT^{*}$. We assume that the adversary knows $(S^{*},\;SK^{*},\;TK^{*},\;PK^{*}).$ The adversary wins the game if
  $${\mathrm{Decrypt}}_{out}\left(PK,CT^{*},TCT^{*},PK^{*}\right)\notin \left\{M^{*},⊥\right\}.$$

The advantage of the adversary A is

$$Adv_{ABE_{out}}^{verify}\left(1^{\lambda }\right)=\mathrm{Pr}\left[A\;wins\right].$$

**Definition** **A2.** (Verifiability) An ABE-VOD scheme is verifiable, if for any polynomial time adversary A, the advantage $Adv_{ABE_{out}}^{verify}\left(1^{\lambda }\right)$ is negligible in the security parameter.

## Appendix B. Review of Li et al.’s Abe-Vod Scheme

Here, we recall the ABE-VOD scheme proposed by Li et al.

- Setup $(1^{\lambda },U).$ Take as input the security parameter $1^{\lambda }$ and the attribute set *U* = $\left\{att_{1},att_{2},\cdots ,att_{l}\right\}$. Generate bilinear group $(p,G_{1},G_{2},e),$ where $G_{1}$ and $G_{2}$ are two multiplicative groups with a prime order p. Choose a random generator $g\in G_{1},$ random elements $h_{1},\cdots ,h_{l}\in Z_{p}^{*}$ and $a,\;\alpha \in Z_{p}^{*}$, computes $y=g^{a}.$ Then generate three collision resistance hash functions
  $$H_{1}:G_{1}\to Z_{p},$$
  $$H_{2}:{\left\{0,1\right\}}^{*}\to G_{1}$$
  and
  $$H_{3}:G_{2}\to {\left\{0,1\right\}}^{m}.$$
  PK = $(G_{1},G_{2},$ g, y, $e{\left(g,g\right)}^{\alpha },$ $H_{1},$ $H_{2},$ $H_{3},$ ${\left\{T_{i}=g^{h_{i}}\right\}}_{i\in U})$ are published as the public parameters. The master secret key msk is α.
- KeyGen(msk,PK,S). To generate private keys for two types of clients (the authorized client and the unauthorized client). If *S* is an attribute set of the authorized client, then the algorithm picks a random value $t_{1}\in Z_{p}^{*}.$ The private key of the authorized client is
  $$SK_{DS}=\left(DS,K=g^{\alpha }y^{t_{1}},K_{0}=g^{t_{1}},{\left\{K_{i}=T_{i}^{t_{1}}\right\}}_{att_{i}\in DS}\right).$$
  If *S* is an attribute set of the unauthorized client, then the algorithm picks a random value $t_{2}\in Z_{p}^{*}.$ The private key for the unauthorized client is
  $$SK_{VS}=\left(VS,KP=g^{\alpha }y^{t_{2}},KP_{0}=g^{t_{2}},{\left\{KP_{i}=T_{i}^{t_{2}}\right\}}_{att_{i}\in VS}\right).$$
- Encrypt$(M,A,\bar{A}).$ Take as input a message $M\in {\left\{0,1\right\}}^{m}$ and two LSSS access structures A = (A,ρ), $\bar{A}$ = $\left(\bar{A},\bar{\rho }\right)$. *A* and $\bar{A}$ are two l×n matrixes. ρ is a map from each row $A_{i}$ of *A* to an attribute ρ(i) and $\bar{\rho }$ is a map from each row $\bar{A_{i}}$ of $\bar{A}$ to an attribute $\bar{\rho }\left(i\right)$. The encryption algorithm first picks a random string $R\in {\left\{0,1\right\}}^{m}$ and two random vectors
  $$\vec{v}=\left(s_{1},v_{12},\cdots ,v_{1n}\right)\in {\left(Z_{p}^{*}\right)}^{n}$$
  and
  $${\vec{v}}^{\prime }=\left(s_{2},v_{22},\cdots ,v_{2n}\right)\in {\left(Z_{p}^{*}\right)}^{n}.$$
  For each row $A_{i}$ of *A*, ${\bar{A}}_{i}$ of $\bar{A}$, it picks $r_{1,i},r_{2,i}\in Z_{p}^{*}$ uniformly at random. Then it computes:
  $$C_{M}=M⊕H_{3}\left(e{\left(g,g\right)}^{\alpha s_{1}}\right),$$
  $$\eta_{1}=H_{1}\left(e{\left(g,g\right)}^{\alpha s_{1}}\right),$$
  $$C_{1}=g^{s_{1}},$$
  $$C_{1,i}=g^{aA_{i}\cdot \vec{v}}T_{\rho (i)}^{-r_{1,i}},D_{1,i}=g^{r_{1,i}}\forall i\in \left\{1,2,\cdots ,l\right\}.$$
  Set $CT_{M}=\left(C_{M},C_{1},{\left\{C_{1,i}\right\}}_{i\in [l]},{\left\{D_{1,i}\right\}}_{i\in [l]}\right).$
  $$C_{R}=R⊕H_{3}\left(e{\left(g,g\right)}^{\alpha s_{2}}\right),$$
  $$\eta_{2}=H_{1}\left(e{\left(g,g\right)}^{\alpha s_{2}}\right),$$
  $$C_{2}=g^{s_{2}},$$
  $$C_{2,i}=g^{a{\bar{A}}_{i}\cdot {\vec{v}}^{\prime }}T_{\bar{\rho }\left(i\right)}^{-r_{2,i}},D_{2,i}=g^{r_{2,i}}\forall i\in \left\{1,2,\cdots ,l\right\}.$$
  Set $CT_{R}=\left(C_{R},C_{2},{\left\{C_{2,i}\right\}}_{i\in [l]},{\left\{D_{2,i}\right\}}_{i\in [l]}\right).$
  $$\sigma_{1}=H_{2}{\left(C_{M},C_{R}\right)}^{\eta_{1}},\sigma_{M}=\left\{\sigma_{1},H_{2}\left(C_{M},C_{R}\right)\right\},$$
  $$\sigma_{2}=H_{2}{\left(C_{M},C_{R}\right)}^{\eta_{2}},\sigma_{R}=\left\{\sigma_{2},H_{2}\left(C_{M},C_{R}\right)\right\}.$$
  The ciphertext $CT=(A,\bar{A},CT_{M},\sigma_{M},CT_{R},\sigma_{R},).$
- Decrypt (SK,S,CT). Take as input the private key SK, an attribute set *S* of the client and a ciphertext CT = $(A,\bar{A},CT_{M},\sigma_{M},CT_{R},\sigma_{R}).$
  - (1) If *S* satisfies the access policy A, then the client is an authorized one and the private key of the client is SK= (DS, $K=g^{\alpha }y^{t_{1}},$ $K_{0}$ = $g^{t_{1}},$ ${\left\{K_{i}=T_{i}^{t_{1}}\right\}}_{att_{i}\in DS}).$ Let *I* = {i:ρ(i)∈S}⊂{1,2,⋯,l}. Then the client is able to compute $\omega_{i}\in Z_{p}^{*}$ for i∈I such that $\Sigma_{i\in I}\omega_{i}A_{i}=\left(1,0,\cdots ,0\right),$ and the client calculates:
    $$X_{M}=\frac{e(C_{1},K)}{\prod_{i\in I}{\left(e\left(C_{1,i},K_{0}\right)e\left(D_{1,i},K_{\rho (i)}\right)\right)}^{\omega_{i}}}=e{\left(g,g\right)}^{\alpha s_{1}},$$
    and $\eta_{1}=H_{1}\left(X_{M}\right).$ After the client checks whether the following equality
    $$e\left(\sigma_{1},g\right)=e(H_{2}(C_{M}\left|\right|C_{R}),g^{\eta_{1}})$$
    holds or not. If it holds, the client calculates
    $$M=C_{M}⊕H_{3}\left(X_{M}\right);$$
    otherwise, the client outputs ⊥.
  - (2) If *S* satisfies the access policy $\bar{A}$, then the client is an unauthorized one and the private key of the client is SK = (VS, $K=g^{\alpha }y^{t_{2}},$ $K_{0}$ = $g^{t_{2}},$ ${\left\{K_{i}=T_{i}^{t_{2}}\right\}}_{att_{i}\in VS}).$ Let *I* = {i:ρ(i)∈S}⊂{1,2,⋯,l}. Then the client is able to compute $\omega_{i}\in Z_{p}^{*}$ for i∈I such that $\Sigma_{i\in I}\omega_{i}\bar{A_{i}}=\left(1,0,\cdots ,0\right),$ and the client calculates:
    $$X_{R}=\frac{e(C_{2},KP)}{\prod_{i\in I}{\left(e\left(C_{2,i},KP_{0}\right)e\left(D_{2,i},KP_{\bar{\rho }\left(i\right)}\right)\right)}^{\omega_{i}}}=e{\left(g,g\right)}^{\alpha s_{2}},$$
    and $\eta_{2}=H_{1}\left(X_{R}\right).$ After the client checks whether the following equality
    $$e\left(\sigma_{2},g\right)=e(H_{2}(C_{M}\left|\right|C_{R}),g^{\eta_{2}})$$
    holds or not. If it holds, the client computes
    $$R=C_{R}⊕H_{3}\left(X_{R}\right);$$
    otherwise, the client outputs ⊥.
- GenTK${}_{out}\left(SK\right).$ Take the private key SK as input. If the client is an authorized one, the private key is SK (DS, $K=g^{\alpha }y^{t_{1}},$ $K_{0}$ = $g^{t_{1}},$ ${\left\{K_{i}=T_{i}^{t_{1}}\right\}}_{att_{i}\in DS}).$ If the client is an unauthorized one, the private key is SK = (VS, $K=g^{\alpha }y^{t_{2}},$ $K_{0}$ = $g^{t_{2}},$ ${\left\{K_{i}=T_{i}^{t_{2}}\right\}}_{att_{i}\in VS}).$ Then the client picks two random values $z_{1},z_{2}\in Z_{p}^{*},$ and the transformation keys are
  $$TK_{DS}=\left(DS,K^{\prime }=K^{1/z_{1}},K_{0}^{\prime }=K_{0}^{1/z_{1}},{\left\{K_{i}^{\prime }=K_{i}^{1/z_{1}}\right\}}_{att_{i}\in DS}\right),$$
  and
  $$TK_{VS}=\left(VS,KP^{\prime }=KP^{1/z_{2}},KP_{0}^{\prime }=KP_{0}^{1/z_{2}},{\left\{KP_{i}^{\prime }=KP_{i}^{1/z_{2}}\right\}}_{att_{i}\in VS}\right),$$
  respectively. The retrieving keys are $RK_{DS}=z_{1}$ and $RK_{VS}=z_{2},$ respectively.
- Transform${}_{out}\left(TK,CT\right).$ Takes as input the ciphertext CT and the transformation key TK. For the authorized client, the transformation key is $TK=TK_{DS}$, and for the unauthorized client, the transformation key is $TK=TK_{VS}$. The transformed is described as follows.
  $$T_{1}^{\prime }=\frac{e(C_{1},K^{\prime })}{\prod_{i\in I}{\left(e\left(C_{1,i},K_{0}^{\prime }\right)e\left(D_{1,i},K_{\rho {\left(i\right)}^{\prime }}\right)\right)}^{\omega_{i}}}=e{\left(g,g\right)}^{\alpha s_{1}/z_{1}},$$
  $$T_{2}^{\prime }=\frac{e(C_{2},KP^{\prime })}{\prod_{i\in I}{\left(e\left(C_{2,i},KP_{0}^{\prime }\right)e\left(D_{2,i},KP_{\bar{\rho }\left(i\right)}^{\prime }\right)\right)}^{\omega_{i}}}=e{\left(g,g\right)}^{\alpha s_{2}/z_{2}}.$$
  Finally, the transformed ciphertext
  $$TCT^{\prime }=\left(C_{M},T_{1}^{\prime },\sigma_{M}\right)$$
  if the attribute set *S* of the user satisfies the access policy A or
  $$TCT^{\prime }=\left(C_{R},T_{2}^{\prime },\sigma_{R}\right)$$
  if the attribute set *S* of the client satisfies the access policy $\bar{A}.$
- Decrypt${}_{out}\left(CT,TCT^{\prime },RK\right).$ Takes as input the ciphertext CT = (A, $\bar{A},$ $CT_{M},$ $\sigma_{M},$ $CT_{R},$ $\sigma_{R}),$ the transformed ciphertext $TCT^{\prime }$ and the retrieving key RK. The retrieving key of the authorized client RK = $RK_{DS}$ = $z_{1}$ and the retrieving key of the unauthorized client RK = $RK_{VS}$ = $z_{2}.$
  - (1) If the attribute set *S* of the client satisfies the access policy A, the client verifies whether
    $$e\left(\sigma_{1},g\right)=e(H_{2}(C_{M}\left|\right|C_{R}),g^{H_{1}\left(T_{1}^{{}^{\prime }z_{1}}\right)})$$
    holds, if it does, then the client outputs
    $$M=C_{M}⊕H_{3}\left(T_{1}^{{}^{\prime }z_{1}}\right);$$
    otherwise, the client outputs ⊥.
  - (2) If the attribute set *S* of the client satisfies the access policy $\bar{A},$ the client verifies whether
    $$e\left(\sigma_{2},g\right)=e(H_{2}(C_{M}\left|\right|C_{R}),g^{H_{1}\left(T_{2}^{{}^{\prime }z_{2}}\right)})$$
    holds, if it does, then the client outputs
    $$R=C_{R}⊕H_{3}\left(T_{2}^{{}^{\prime }z_{2}}\right);$$
    otherwise, the client outputs ⊥.
